# Pinin facilitated proliferation and metastasis of colorectal cancer through activating EGFR/ERK signaling pathway

**DOI:** 10.18632/oncotarget.8738

**Published:** 2016-04-15

**Authors:** Zhigang Wei, Wenhui Ma, Xiaolong Qi, Xianjun Zhu, Yutian Wang, Zhuoluo Xu, Jun Luo, Da Wang, Weihong Guo, Xiaomei Li, Sainan Xin, Jiang Yu, Guoxin Li

**Affiliations:** ^1^ Department of General Surgery, Nanfang Hospital, Southern Medical University, Guangzhou, China; ^2^ Department of Pathology, Nanfang Hospital, Southern Medical University, Guangzhou, China

**Keywords:** colorectal cancer, PNN, EGFR/ERK, metastasis

## Abstract

Increasing emphasis has been put on the influence of desmosome related proteins on progress of colorectal cancer (CRC). Pinin (PNN) is a desmosome-associated molecule that has been reported its overexpression could increase desmoglein 2 (DSG2) and E-cadherin (E-ca) levels. However, it was documented that DSG2 and E-ca had opposite functions in CRC. Thus, we attempted to elucidate function and mechanism of PNN in CRC. Herein, we revealed that overexpression of PNN was significantly correlated with the aggressive characteristics and indicated poor overall survival of CRC patients. In addition, the proliferation, invasion *in vitro*, and tumorigenic growth, metastasis *in vivo* were also promoted by the up-regulation of PNN. It was also verified that up-regulation of PNN increased the expression of DSG2 and activated the EGFR/ERK signaling pathway. Our findings suggested that PNN, as a valuable marker of prognosis, has important influence on the progression of CRC.

## INTRODUCTION

Colorectal cancer (CRC) ranked the third of cancer incidence and the fourth of cancer death in 2013 [1]. A large number of deaths occurred in CRC patients owing to no effective approach for the cure of advanced CRC [2, 3]. Although an increasing number of oncogenes have been reported to be responsible for the development of CRC, such as S100P [4], K-Ras [5] and BRAF [6], efforts are still needed to clarify the molecular mechanisms of migration and invasion of advanced CRC.

Pinin (PNN), a 140 kDa phosphoprotein, was first identified and characterized as a desmosome-associated molecule [7]. It is not integral to the desmosome, but associated with only mature one [8]. Desmosomes are intercellular junctions that tether intermediate filaments (IF) to the plasma membrane [9]. The presence of PNN with the desmosome is correlated to highly organized, perpendicular bundles of keratin filaments, and primarily stabilizes the desmosome-IF association and reinforces the epithelial cells adhesion [8]. Furthermore, both endogenous and exogenous PNNs are not only in cytomembrane and cytoplasm, but also diffusely throughout nucleoplasm [10].

Compelling evidences suggested that PNN played a role in pre-mRNA splicing by decreasing the use of distal splice sites [11, 12]. This might be as a result of the interaction of PNN with serine-arginine (SR)-rich proteins, such as SRp75, SRm300 and SRrp130 [13]. Moreover, PNN appears to associate preferentially with spliced mRNA and involve in mRNA export [14]. It was also noted that down-regulation of PNN led to the suppression of cadherin superfamily members (E-cadherin (E-ca), desmoglein-2 (DSG2), desmoplakin) and consequently induced a loss of epithelial adhesion [10]. The repression of E-ca could be relieved though the interaction of transcriptional co-repressor CtPB and PNN [15]. Recently, studies indicated that different localizations of PNN, like β-catenin, might play diverse roles in tissue remodeling and tumor progression [16, 17]. Furthermore, the potential role of PNN as a tumor suppressor was presented based on its genetic locus and methylated CpG islands [18]. Interestingly, E-ca as a tumor suppressor [19–21] and DSG2 as a potential oncogene [22] in CRC could be both increased by PNN overexpresion, which suggested the different mechanisms of PNN in CRC patients. The study aims to evaluate the role of PNN in growth, migration and invasion of CRC both *in vitro* and *in vivo*. In addition, the predictive value of PNN for CRC patients was also investigated.

## RESULTS

### PNN is overexpressed in various types of cancers and associated with tumor progression

**Figure 1:**
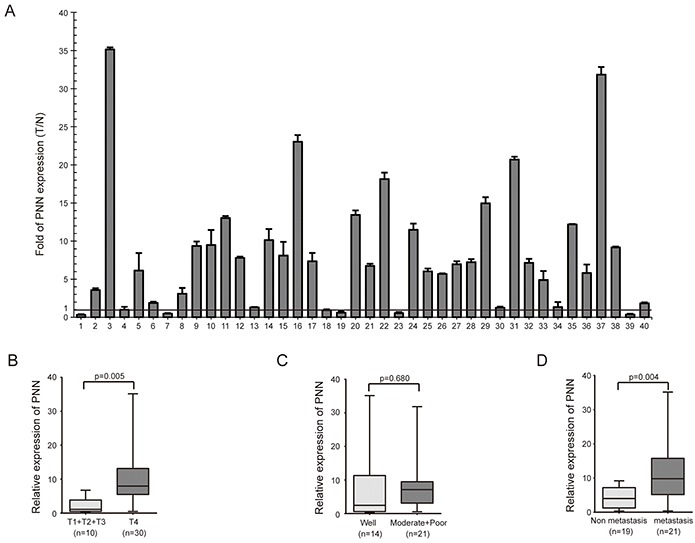
PNN is highly expressed in CRC and correlated with T classification and metastasis **A.** PNN expression in 40 CRC biopsies and their matched adjacent normal tissues. **B, C.** and **D.** the relationship of PNN and T classification, differentiation andmetastasis of CRC.

The expression of PNN was detected by real-time PCR in 40 CRC biopsies and their matched adjacent normal tissues. mRNA levels of PNN showed a higher expression in 70% (28/40) CRC samples than normal ones (Figure 1A). The levels of PNN were analyzed to further investigate its clinicopathological significance. Correlation analysis showed that a high-level expression of PNN in CRC was significantly associated with a more aggressive T classification (Figure 1B, p=0.005) and metastasis (Figure 1D, p=0.004). However, there was no visible correlation between PNN and CRC differentiation (Figure 1C, p=0.680).

**Figure 2:**
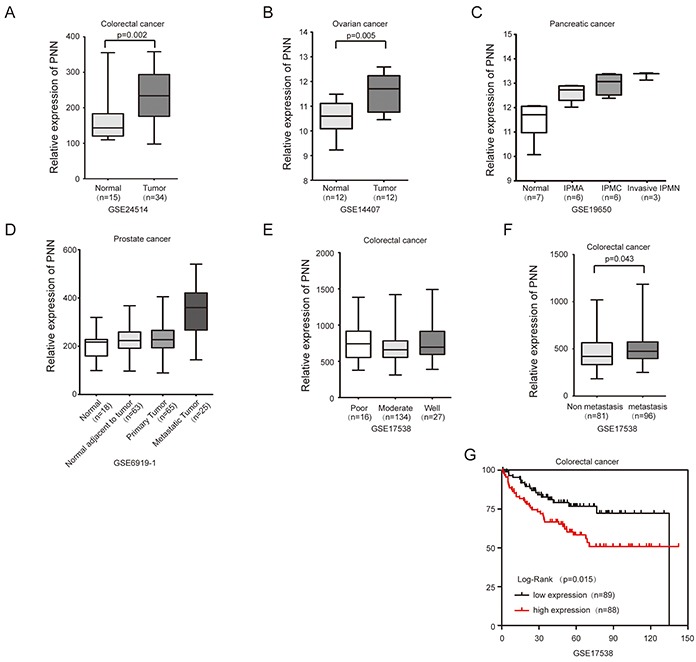
PNN overexpressed in tumors and is associated with the prognosis of CRC **A.** PNN in CRC and matched normal tissues. **B.** PNN in ovarian cancer and matched normal tissues. **C** and **D.** PNN in the progression of pancreatic cancerand prostate cancer. **E, F** and **G.** the relationship of PNN and differentiation, metastasisand overall survival of 177 CRC patients. IPMN= intraductal papillary mucinous neoplasm IPMA= IPMN-adenoma. IPMC= IPMN-carcinoma.

We further analyzed PNN expression in CRC and other tumor tissues based on GEO database. The level of PNN was significantly higher in CRC (Figure 2A, p=0.002) and ovarian cancer (Figure 2B, p=0.005) compared to their adjacent normal tissues. Besides, PNN was associated with tumor progression of pancreatic cancer (Figure 2C, p<0.001) and prostate cancer (Figure 2D, p<0.001). According to a large cohort of 177 CRC tissues, levels of PNN were not correlated with the degree of differentiation (Figure 2E, p=0.129), but significantly increased in metastatic CRC tissues (Figure 2F, p=0.043). Kaplan-Meier analysis revealed that a high-level PNN was associated with a short overall survival of patients with CRC (Figure 2G, p=0.015).

### PNN is associated with proliferation, migration and invasion of CRC *in vitro*

**Figure 3:**
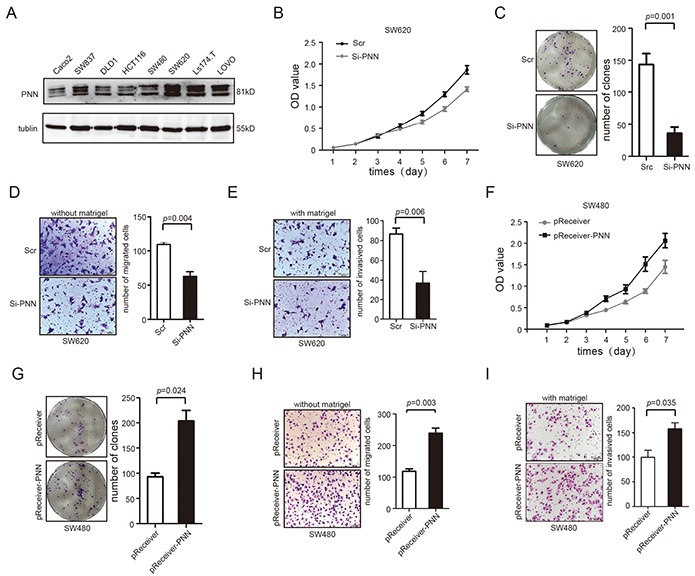
PNN level is related to proliferation, migration and invasion of CRC in vitro **A.** PNN in 8 CRC cell lines analyzed by Western blot. **B** and **C.** Down-regulation of PNN inhibited proliferation of SW620 determined by CCK-8 and colony formation assays. **D** and **E.** Down-regulation of PNN inhibited migration and invasion of SW620 determined by transwell assays. **F.** and **G.** Ectopic expression of PNN stimulated proliferation of SW480 determined by CCK-8 and colony formation assays. **H** and **I.** Ectopic expression of PNN promoted migration and invasion of SW480 determined by transwell assays. Error bars represent mean±SD.

Endogenous expression of PNN was detected by western-blotting in 8 CRC cell lines. A high expression level of PNN was observed in SW620, SW837, Ls174.T and LOVO and its expression in SW480, Caco2, DLD1 and HCT116 was relatively low (Figure 3A). Next, SW480 and SW620 cell lines were selected for further experiments.

We knocked down the PNN expression in SW620 by 3 alternative SiRNAs and scrambled SiRNA(Scr) was transfected as a negative control. The results showed that PNN levels were dramatically knocked down by the third SiRNA (Supplementary Figure S1A and S1B). We then examined the effect of PNN on cellular proliferation. CCK-8 and colony formation assays revealed that down-regulation of PNN significantly inhibited the proliferation rate of SW620 cells compared to negative control (Figure 3B, p<0.001 and Figure 3C, p=0.001). Moreover, we determined the rates of migration (transwell chambers covered without matrigel) and invasion (transwell chambers covered with matrigel) in cells transfected by SiRNA. As shown in Figure 3D and Figure 3E, both migration (p=0.004) and invasion (p=0.006) were obviously inhibited in PNN knock-down cells. Conversely, pReceiver-PNN was transfected into SW480 to elevate the expression of PNN and pReceiver vector was simultaneously transfected as a control (Supplementary Figure S1C and S1D). Exogenetic overexpression of PNN in SW480 caused an increase of cell viability (Figure 3F, p<0.001 and Figure 3G, p=0.024) and markedly promoted the migration and invasion (Figure 3H, p=0.003 and Figure 3I, p=0.035).

### PNN is correlated with growth and metastasis of CRC *in vivo*

**Figure 4:**
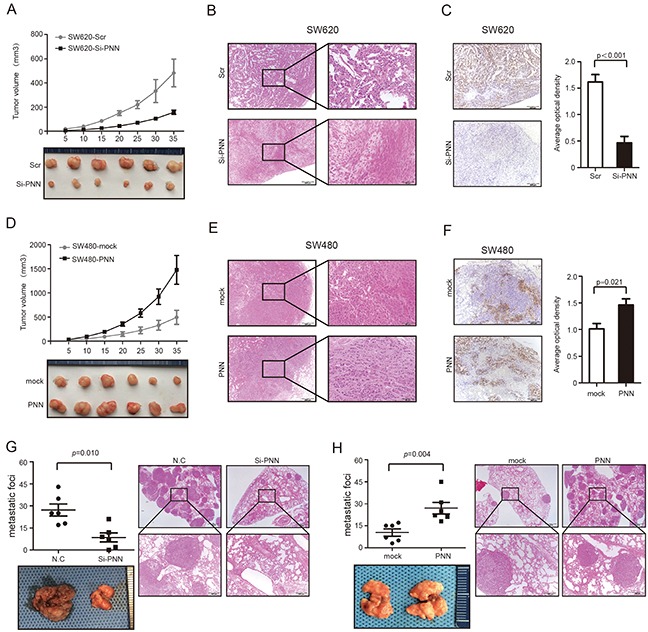
PNN is related to tumor growth and metastasis of CRC *in vivo* **A.** Growth curve of subcutaneous injection of SW620/Scramble and SW620/shPNN in nude mice (n=6 per group). **B** and **C.** Representative tumors of SW620/Scramble and SW620/shPNN analyzed by H&E and Ki-67 staining. **D.** Growth curve of subcutaneous injection of SW480/Vector and SW480/PNN in nude mice (n=6 per group). **E.** and **F.** Representative tumors of SW480/Vector and SW480/PNNanalyzed by H&E and Ki-67 staining. AOD ratio (average optical density, AOD = 100×IOD/AOI) was used to represent the expression level of Ki-67 (n=6 per group). **G.** Lung metastatic nodules after 50 days of intravenous tail injection of SW620/Scrambled and SW620/shPNN. **H.** Lung metastatic nodules after 50 days intravenous tail injection of SW480/Vector and SW480/PNN. Data presented as mean± SD.

Stable PNN interference cells and the negative control (scrambled SiRNA) cells were injected subcutaneously into nude mice and tumor volume was supervised. Notably, the growth of CRC was significantly suppressed in nude mice that were injected with PNN down-regulation SW620 (Figure 4A, p<0.001 and Figure 4B). According to immunohistochemistry (IHC) staining, tumors transfected with scrambled SiRNA in the control group displayed much higher Ki-67 indexes than the ones in PNN interference group (Figure 4C, p<0.001). In contrast, overexpression of PNN in nude mice promoted the SW480 growth rate (Figure 4D, p<0.001 and Figure 4E) and also increased the Ki-67 indexes (Figure 4F, p=0.021). To explore the role of PNN in CRC metastasis, cells were injected intravenously into the tails of nude mice and the number of lung metastatic nodules was counted. We found that down-regulation of PNN significantly decreased metastatic foci in the lung (Figure 4G, p=0.010) and overexpression of PNN increased metastatic nodules (Figure 4H, p=0.004).

### PNN activates EGFR/ERK signaling pathways through up-regulating DSG2

**Figure 5:**
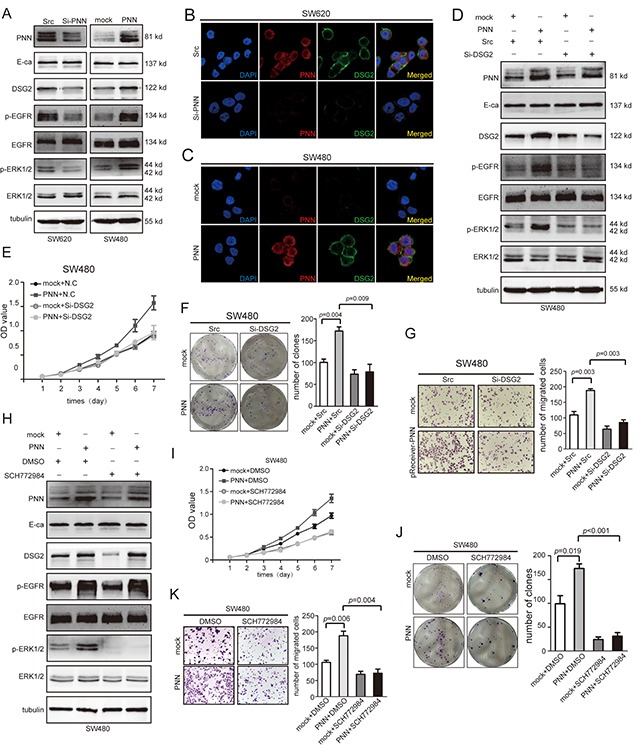
DSG2 and ERK inhibitor reverses the effect of PNN on proliferation and migration *in vitro* **A.** Indicated proteins of SW480/Vector and SW480/PNN, SW620/N.C and SW620/Si-PNN analyzed by Western blot. **B** and **C.** PNN and DSG2 of SW480/Vector and SW480/PNN, SW620/N.C and SW620/Si-PNN analyzed by IFC. **D.** Indicated proteins of SW480/Vector and SW480/PNN combined with treatment of Si-DSG2 analyzed by Western blot. **E** and **F.** Combined effect of PNN and DSG2 on SW480 proliferationdetermined by CCK-8 and colony formation assays. **G.** Combined effect of PNN and DSG2 on SW480 migration determined by transwell assays. **H.** Indicated proteins of SW480/Vector and SW480/PNN combined with ERK blocker. **I** and **J.** Effect of ERK blocker on proliferation of SW480/Vector and SW480/PNN determined by CCK-8 and colony formation assays. **K.** Effect of ERK blocker on migration of SW480/Vector and SW480/PNN determined by transwell assays. Error bars represent mean±SD.

Both DSG2 and E-ca expressed in colorectal epithelial cells and could be stimulated by PNN in corneal epithelial cells [10]. Interestingly, down-regulation of PNN in SW620 suppressed the expression of DSG2 but not E-ca (Figure 5A left). Next, we evaluated the expression of total and phosphorylation of EGFR and ERK1/2 through which DSG2 could affect cell proliferation [22]. The results showed that down-regulation of PNN suppressed the phosphorylation of EGFR and ERK1/2 and had no effect on total EGFR and ERK1/2 (Figure 5A left). On the contrary, PNN overexpression could increase the level of DSG2 and phosphorylation of EGFR and ERK1/2 (Figure 5A right). From the results of immunofluorescence (IFC) assay, the level of DSG2 was suppressed when SW620 were transfected with Si-PNN (Figure 5B). Conversely, DSG2 was elevated when PNN was promoted in SW480 (Figure 5C). However, phosphorylation levels of EGFR and ERK1/2 were suppressed when PNN-overexpressed SW480 was transfected with Si-DSG2 (Figure 5D). In order to elucidate the relationship between PNN and DSG2, stably ectopic PNN-overexpressed SW480 cells were transfected with Si-DSG2 and scrambled SiRNA to evaluate the proliferation, migration and invasion *in vitro*. It is noted that knocking down the expression of DSG2 suppressed the proliferation (Figure 5E mock+Scr vs PNN+Scr p<0.001, PNN+Scr vs PNN+Si-DSG2 p<0.001 and Figure 5F mock+Scr vs PNN+Scr p=0.004, PNN+Scr vs PNN+Si-DSG2 p=0.009), migration (Figure 5G) of CRC cells in spite of PNN overexpression.

### ERK inhibitor reverses the biological functions of PNN

SCH772984, an ERK inhibitor, was added into PNN-overexpressed and normal SW480 cells, respectively. As a result, when PNN overexpressing cell was treated with SCH772984, the phosphorylation of ERK1/2 was markedly inhibited without suppression of DSG2 and p-EGFR (Figure 5H). Besides, the level of p-ERK1/2 was not inhibited by DMSO that used as a solvent for SCH772984. Furthermore, CCK-8 and colony formation assays indicated that elevated proliferation rate of SW480 cells by PNN could be suppressed by SCH772984 (Figure 5I mock+DMSO vs PNN+DMSO p<0.001, PNN+DMSO vs PNN+SCH772984 p<0.001 and Figure 5J). We also noticed that abilities of migration and invasion *in vitro* were inhibited by SCH772984 (Figure 5K).

### High-level PNN is associated with an aggressive CRC and poor overall survival

**Figure 6:**
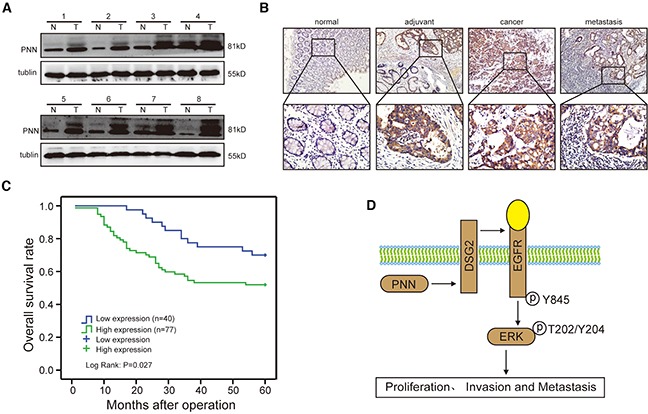
PNN is overexpessed in CRC and associated with an aggressive and poor survival **A.** PNN expression in 8 paired human CRC biopsies and matched normal mucosa analyzed by Western-blot. **B.** a representative case of PNN expression stained by IHC. **C.** overall survival of 117 CRC patients stratified by PNN expression. **D.** the potential role of PNN in CRC and diagrammatic sketch.

The expressions of PNN of 8 CRC tissues and matched normal tissues were detected by western blot. PNN was increased significantly in CRC tissues compared with adjacent normal mucosa (Figure 6A). Moreover, results of IHC analyses revealed that PNN was overexpressed in tumor tissue and metastasis lymph node compared to the adjuvant tissue and normal mucosa (Figure 6B). In a large cohort of 117 CRC patients, the intensity of PNN staining was recorded on a scale of 0 (no staining), 1 (weak staining), 2 (moderate staining) and 3 (strong staining). 65.8% (77/117) CRC tissues showed higher expression of PNN compared to matched normal tissues. In addition, a high-level PNN was significantly correlated with a more aggressive T stage (p=0.040), lymph node metastasis (p=0.001) and distance metastasis (p=0.018) (Supplementary Table S1). Kaplan-Meier analysis further revealed that a high expression of PNN was associated with a short overall survival of patients with CRC (Figure 6C, p=0.027).

## DISCUSSION

Metastasis-associated genes determine the abilities of cancer cells to invade surrounding tissues and facilitate the tumor dissemination [23]. It has been documented that alternation of desmosomal composition was associated with the abilities of tumor cell invasiveness and metastasis [24, 25]. Nevertheless, different desmosomal components might have varying influence on tumor progression. Desmocollin-2 (DSC2) and DSG2, two transmembrane cell adhesion proteins of desmosomes, were reported with opposite functions in tumor progression. Low-expression of DSC2 was found in CRC [26, 27] and was associated with activation of Akt/β-catenin signaling [28], while loss of DSG2 suppressed the EGFR/ERK signaling in CRC [22]. Furthermore, DSG2 was also supposed to be an oncogene in prostate cancer [29]. Herein, we elucidated the biological function of another desmosome-associated molecule, PNN, both *in vitro* and *in vivo*, and also evaluated the prognostic value of PNN for CRC patients.

PNN was previously supposed as a tumor suppressor (e.g. in renal cell carcinomas) by analysis of genetic location and methylation of CpG islands [18]. However, in this study, when the expression of PNN in CRC biopsies and matched normal tissue were evaluated, we noticed that higher mRNA expressions of PNN were detected in CRC biopsies. Besides, PNN was also elevated in some other cancers. Moreover, overexpression of PNN was significantly associated with an aggressive tumor phenotype and a poor prognosis according to our study and the results of GEO database. Thus, it indicated that PNN might function as a CRC promoter. In addition, we found that PNN promoted the proliferation and invasion of CRC *in vitro*, and also facilitated tumor growth and metastasis *in vivo*. Consistent with this, it has been previously reported that PNN was essential for survival of mouse embryos and the breast carcinoma cell line MCF-7 [30]. Considering that the animal model artificial pulmonary metastasis by tail vein injection in nude mice, which already lacks parts of immune microenvironment, it would be ideal to have used murine cancer cell lines in immunocompetent mice. Besides, the potential tumor-suppression function of PNN was also supposed based on the association of PNN and epithelial cell-cell adhesion [8, 31]. Whether the different localizations of PNN in varying types of cells determine distinct functions have yet to be further elaborated.

Recently, it was reported that DSG2 and E-ca could be induced by PNN in human corneal epithelial cells [10, 15]. However, we found that only DSG2 was promoted in PNN-overexpressed CRC cells. It was suggested that DSG2 promoted the proliferation of CRC through activating EGFR/ERK signaling pathway [22]. In this study, EGFR/ERK signaling pathway was also sensitized to the up-regulation of PNN. Moreover, the absence of DSG2 could reverse the effect of PNN overexpression on CRC proliferation andmigration, which further supported the accelerated role of DSG2 in colonic epithelium. These findings were consistent with earlier studies in which an increased expression of DSG isoforms in other carcinomas was demonstrated [32–35]. Moreover, the data presented herein provided the first direct evidence that PNN and DSG2 showed consistent functions in the activation of EGFR/ERK signaling pathway, and highlighted a mechanistic interplay between desmosomal cadherin and desmosome-associated molecule. However, the interaction of PNN and DSG2 was not direct according to the binding analysis by Co-IP (Supplementary Figure S2). The specific regulatory mechanism between PNN and DSG2 need to be further elucidated. We also clarified that the activated EGFR/ERK pathway by PNN could also be inhibited by ERK blocker. This result provided solid evidence for the influence of PNN on EGFR/ERK signaling pathway. Impressively, a high-level PNN always showed up in aggressive tumors and was correlated with a poor overall survival of CRC patients. Thus, we proposed a model (Figure 6D) in the study that up-regulation of PNN activated EGFR/ERK signaling pathway through DSG2 and consequently promoted the proliferation, invasion and metastasis of CRC.

## MATERIALS AND METHODS

### Tissue specimens

40 biopsies of CRC tissues and matched adjacent noncancerous mucosa tissues were obtained from patients who were diagnosed with CRC and then underwent elective surgery in Nanfang Hospital, Southern Medical University, Guangzhou, China. Paraffin-embedded, archived CRC samples were obtained from 117 patients diagnosed as CRC between January 2005 and December 2008 at Nanfang Hospital, Southern Medical University. Medical records of the 117 patients included age, gender, tumor histology, T stage, differentiation, lymph node metastases, distant metastasis and survival. All data were collected and preserved by SCLASS system in Department of General Surgery, Nanfang Hospital, Southern Medical University. The protocol was approved by the ethics committee of Nanfang Hospital, Southern Medical University.

### RNA extraction and quantitative PCR

Total RNA was extracted with TRIzol reagent (TaKaRa, Dalian China) and cDNA was synthesized from 5ng of total RNA using the mRNA reverse transcription kit (TaKaRa, Dalian China). The qRT-PCR Detection Kit (TaKaRa, Daian China) was used for quantitative detection of mRNA.

### PNN expression in GEO database

Expression of PNN in CRC and other tumor tissues was analyzed based on the GEO database The expression profiles by array of GSE24514, GSE14407, GSE19650, GSE6919-1, GSE17538 were downloaded from Gene Expression Omnibus database (GEO, http://www.ncbi.nlm.nih.gov/geo/) in the National Center for Biotechnology Information (NCBI). Specimens were analyzed using oligonucleotide array analysis.

### Cell culture and transfection

Eight CRC cell lines (Caco2, LOVO, DLD1, SW480, SW620, HCT116, SW837, and Ls174.T) were cultured in RPMI 1640 (HyClone) supplemented with 10% heat-inactivated FBS (HyClone). Cells were purchased from the American Type Culture Collection and maintained at the Department of Pathology, Southern Medical University. The small interference RNAs (SiRNAs) and the scrambled SiRNA (N.C) of PNN and DSG2 were purchased from Ruibo (Ruibo, Guangzhou, China) and PNN overexpression plasmid pReceiver-Lv121-PNN and negative control plasmid pReceiver-Lv121CT-vector were purchased from GeneCopoeia (GeneCopoeia, Rockville, USA) that were transfected instantaneous into CRC cells using Lipofectamine 2000 reagent (Invitrogen, Foster, USA). Lentivirus particles were harvested 48h after pReceiver-Lv121-PNN (PNN) and pReceiver-Lv121CT-vector (mock) transfection with the packaging plasmid pRSV/pREV, pCMV/pVSVG and pMDLG/pRRE into 293FT cells using Lipofectamine 2000 reagent (Invitrogen, Foster, USA). The enrolled SiRNA was cloned into pSuper-retro-puro for deletion of PNN. Infectious virus was produced by transfecting retroviral vectors and the pIK packaging vector into 293FT cells. CRC cells were infected with recombinant lentivirus-transducing units plus 8 mg/ml Polybrene (Sigma, Missouri, USA).

### Cell viability assay

Cells were seeded in 96-well plates at initial density of (1×10^3^ per well). 100ul complete medium mix with 10ul CCK-8 solution (Kegene, Nanjing, China) was added to each well at each time point and cultured for 2 h at 37°C. The absorbance (OD value) was measured at 570 nm, with 655 nm as the reference wavelength. All experiments were performed in triplicates.

### Colony formation assays

Cells were trypsinized and plated on 6-well plates (300cells/well) and cultured for 2 weeks. The colonies were stained with giemsa staining for 30min after fixation with 4% paraformaldehyde for 15min. The number of colonies defined as >50 cells/colony were counted. Three independent experiments were performed.

### Cell migration and invasion assay

The transwell chambers were rehydrated with RPMI 1640 (serum-free) for 2 hours at 37°C. Complete medium with was added to the lower compartment as the chemotactic factor. Then 1.5×10^5^ cells in serum-free medium were added to the upper compartment of the chamber. After 48 hours incubation, the nonmigrated cells were removed with a cotton swab. Cells that had migrated through the membrane and stuck to the lower surface of the membrane were stained with hematoxylin and counted under a light microscope in 5 random visual fields (200×). For invasion assay, the upper compartment of the chambers were added with matrigel. Each experiment was repeated 3 times.

### Xenograft model *in vivo*

SW620 and SW480 were engineered using a lentiviral-based system to stably express low-PNN and high-PNN, respectively. A 200-μl suspension of CRC cells at a concentration of 1×10^7^ cells/ml was injected subcutaneously into the left flank or right flank of nude mice (n=6 per group). Tumor volume was measured with calipers after each time of injection. For tail injection, a 200-μl suspension of CRC cells at a concentration of 1×10^7^ cells/ml was injected intravenously into the tails of nude mice (n=6 per group). Pulmonary metastases were detected by H&E staining and quantified by counting metastatic lesions in each section.

### Western blot

Protein lysates were prepared, subjected to SDS-PAGE, transferred onto PVDF membranes and blotted according to standard methods, using anti-PNN, anti-DSG2, anti-E-ca, anti-EGFR, anti-p-EGFR (Abcam, Boston, USA), anti-ERK1/2, anti-p-ERK1/2, (Cell Signaling Technology, MA, USA). An anti-α-tubulin (Sigma, MO, USA) monoclonal antibody was used as a loading control.

### Immunofluorescence (IFC)

Cells were fixed in 4% paraformaldehyde for 15 minutes at room temperature and permeabilized with 1% Triton X-100 for 10 minutes on ice. Slides were incubated in anti-PNN and DSG2 (Abcam, Boston, USA) overnigt at 4°C, an Alexa Fluor-conjugated secondary antibody (Origene, Beijing, China) for 1 hour at 37°C, and nuclei were counterstained with DAPI (Beyotime, Nanjing, China). Confocal dishes were viewed using a laser confocal microscope (Olympus, Tokyo, Japan).

### Immunohistochemistry (IHC)

Tissue sections were incubated with moloclonal mouse antibody against PNN (Abcam, Boston, USA) at dilutions of 1:70 overnight at 4°C. For negative controls, the anti-PNN antibody was replaced with normal nonimmune serum. The cells at each intensity of staining were recorded on a scale of 0 (no staining), 1 (weak staining = light yellow), 2 (moderate staining = yellowish brown), and 3 (strong staining = brown). An intensity score of ≥2 with at least 50% of malignant cells with positive PNN staining was used to classify tumors with high expression, and <50% of malignant cells with nuclear staining or <2 intensity score classified tumors with low expression of PNN. Mouse anti-Ki-67 monoclonal antibody (Abcam, Boston, USA) was used to evaluate the Ki-67 labeling index. The Ki-67 index was assessed in the detected area included integrated optical density (IOD) and area of interest (AOI) by Image-Pro Plus (IPP).We used the AOD ratio (average optical density, AOD = 100×IOD/AOI) to represent the expression level.

### Statistical analysis

Statistical analysis was performed using a SPSS software package (SPSS Standard version 16.0, SPSS Inc.). Data *in vitro* and *in vivo* were presented as mean± SD. The data of CCK-8 and tumor volumes *in vivo* were analyzed by factor analysis and one-way ANOVA and Factorial Analysis. Independent *t* test was used to analyze the results of colony formation, migration and invasion assay. Numbers of metastasis lesions were analyzed by Mann-Whitney U test. For the result of tissue biopsies, differences between variables were assessed by the Chi-square test or Fisher's exact test. A log rank test was used to compare different survival curves. P <0.05 was considered statistically significant.

## SUPPLEMENTARY FIGURES AND TABLE



## Abbreviations

CRC: colorectal cancer
PNN: Pinin
IF: intermediate filament
SR: serine-arginine
E-ca: E-cadherin
DSG: desmoglein
SCLASS: Southern China Laparoscopic colorectal Surgery Study
H&E staining: haematoxylin and eosin staining
IFC: immunofluorescence
IHC: immunohistochemistry
EGFR: epidermal growth factor receptor
ERK: extracellular signalregula ted kinases
DMSO: dimethyl sulphoxide
DSC: desmocollins.
