# Supplementary material for: Pinin facilitated proliferation and metastasis of colorectal cancer through activating EGFR/ERK signaling pathway
Source: Oncotarget. 2016 Apr 15;7(20):29429–39. doi: 10.18632/oncotarget.8738 (PMC5045407; doi:10.18632/oncotarget.8738)
Supplement: Supplementary file 1 [file oncotarget-07-29429-s001.pdf]

SUPPLEMENTARY FIGURES AND TABLE

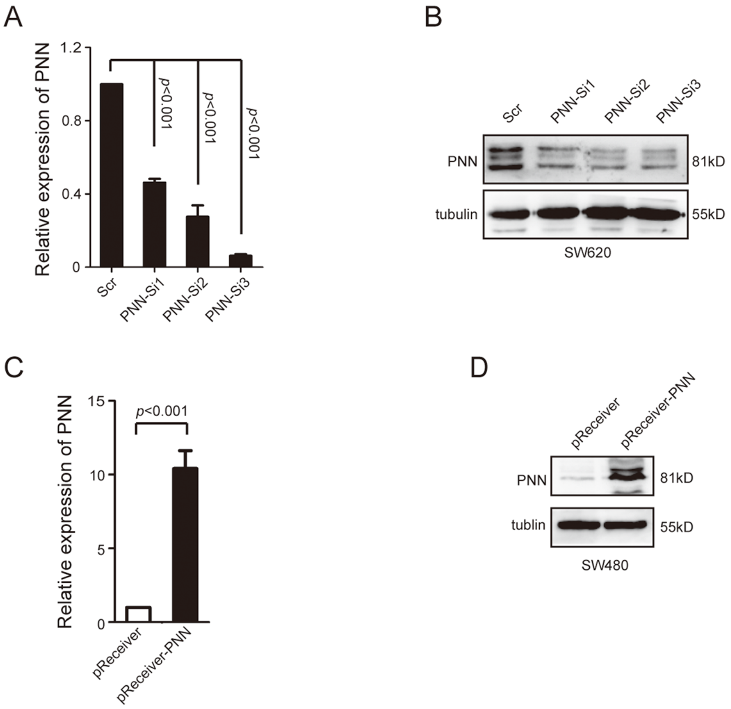

Supplementary Figure S1: Expression of PNN under the interference of PNN-SiRNA and pReceiver-PNN. **A** and **B.** Interfering efficiency of 3 SiRNAs targeting PNN in SW620 by real-time PCR and Western blot. **C** and **D.** Overexpressed efficiency of pReceiver-PNN in SW480 by real-time PCR and Wetern blot. Error bars represent mean±SD.

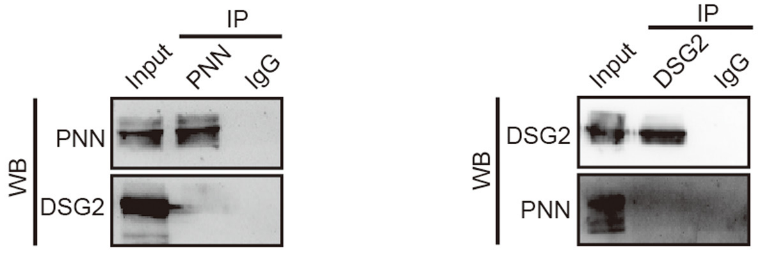

Supplementary Figure S2: Binding analysis of PNN and DSG2 was examined by co-immunoprecipitation (co-IP) followed by Western blotting.

Supplementary Table S1: Clinicopathological characteristics of patients with colorectal cancer

Valuables	PNN			p value
	All cases (n=117)	Low expression (n=40) (%)	High expression (n=77) (%)	
Sex				
Male	74	25 (33.8)	49 (66.2)	1.000
Female	43	15 (34.9)	28 (65.1)	
Age				
>50.2	59	21 (35.6)	38 (64.4)	0.846
<50.2	58	19 (32.8)	39 (67.2)	
Histological type				
Adenocarcinoma	101	36(35.6)	65 (64.4)	0.572
Mucinous adenocarcinoma	16	4 (25.0)	12 (75.0)	
Differentiation				
Well	27	10 (37.0)	17 (63.0)	0.696
Moderate	58	21 (36.2)	37 (63.8)	
Poor and undifferentiated	32	9 (28.1)	23 (71.9)	
T Classification				
T1+T2	20	11 (55.0)	9 (45.0)	0.040
T3+T4	97	29 (29.9)	68 (70.1)	
Lymph node metastasis				
Absent (N0)	50	26 (52.0)	24 (48.0)	0.001
Present (N1-2)	67	14 (20.9)	53 (79.1)	
Distant metastasis				
Absent (M0)	102	39 (38.2)	63 (61.8)	0.018
Present (M1)	15	1 (6.7)	14 (93.3)	
